# Return to Work and Sports After Tibial Plateau Fracture Treatment: Are There Factors Associated with Faster Recovery?

**DOI:** 10.3390/jcm14217802

**Published:** 2025-11-03

**Authors:** Tobias Resch, Lea Faber, Frederik Aasen-Hartz, Philipp Zehnder, Ahmed Ellafi, Peter Biberthaler, Frederik Greve

**Affiliations:** 1 Department of Trauma Surgery, TUM Universitätsklinikum, Klinikum Rechts der Isar, Technical University of Munich, Ismaninger Str. 22, 81675 Munich, Germany; 2 Department of Knee-, Hip-, Shoulder-, and Elbow Surgery, FIFA Medical Centre of Excellence, Schön Klinik München Harlaching, 81547 Munich, Germany

**Keywords:** tibial plateau fracture, return to work, return to sport, rehabilitation, clinical outcomes, surgery, conservative treatment

## Abstract

**Background:** The aim of this study was to separately assess return to work (RTW) and return to sports (RTS) rates and timelines following surgical and conservative treatment of tibial plateau fractures (TPF). A secondary objective was to identify factors associated with faster recovery. **Methods:** All patients with TPF treated at a single level I trauma center between 1 January 2008 and 31 December 2016 were retrospectively reviewed. Standardized questionnaires were used to evaluate pre- and postoperative work and sports activity. Subgroup and correlation analyses were performed to investigate the influence of demographic and treatment-related factors on RTW and RTS duration. **Results:** A total of 105 patients were included, of whom 85% (*n* = 89) received surgical treatment and 15% (*n* = 16) were treated conservatively. RTW was achieved by 100% of surgically treated and 93% of conservatively treated employed patients, with a mean duration of 11.3 ± 9.5 weeks and 6.5 ± 4.2 weeks, respectively. RTS was achieved by 85% of surgically treated and 86% of conservatively treated previously active patients, occurring after a mean of 22.1 ± 17.9 weeks and 12.2 ± 8.8 weeks, respectively. Male sex, lower fracture complexity, absence of external fixation, and shorter operative times were associated with faster recovery. A general shift toward low-impact and recreational sports and a reduction in sport types and weekly training sessions were observed. **Conclusions:** Independent of the treatment modality, high RTW and RTS rates are observed within six months following TPF. The identified factors may help guide patient counseling and improve individual rehabilitation planning.

## 1. Introduction

With a frequency of approximately 1% of all fractures and an annual incidence of around 10.3 per 100,000 patients, tibial plateau fractures (TPF) are rare but serious injuries to the knee joint. [1]. There are two main patient groups affected: 1. young and active patients suffering from high-energy trauma like traffic and sports accidents, and 2. elderly patients with osteoporosis and other comorbidities experiencing low-energy trauma, mainly falls from low height [1,2].

Especially for the first-mentioned group, a timely return to work (RTW) and sports (RTS) is a top priority. The proportion of patients who do not fully return to their previous profession is reported between 8% and 73%, which underlines the relevance of TPF as a potentially life-changing injury [3,4,5,6]. Between 36% and 93% of patients are able to return to some kind of sporting activity [5,7,8,9,10,11]. However, many patients fail to return to their pre-injury level of activity, often engaging in sports at a lower intensity and frequency. There is a noticeable shift towards low-impact activities and away from high-impact sports, with professional athletes frequently facing the end of their careers [7,8,10,11]. In contrast to these findings, patients with TPF often have high expectations for their treatment outcomes and tend to overestimate the prognosis of their injury [12]. To avoid patient frustration and ensure adherence to therapy, adequate counseling is essential. However, the limited number of studies on this topic often report RTW and RTS rates as secondary findings, without providing detailed information on the intensity and extent of activities. Additionally, timelines are rarely documented, and outcomes following conservative treatment remain largely unreported [4,5,13].

Thus, this study aims to separately analyze the RTW and RTS rates and timelines after surgical and conservative treatment of TPF. Additionally, potential factors associated with a timely return to work and sports should be examined.

## 2. Materials and Methods

### 2.1. Patients

This retrospective cohort study received approval from the ethical committee of the Technical University of Munich (No: 2023-14-S-NP) and was conducted in compliance with the ethical guidelines of the 1964 Declaration of Helsinki and its later amendments. All patients who underwent treatment for TPF at a single level I university trauma center between 1 January 2008 and 31 December 2016 were screened for eligibility. Recruitment was carried out through invitation letters, and informed consent was obtained from all participants.

Patients were excluded if they were younger than 18 years, did not reside in Germany, lacked sufficient German skills, refused to participate in the study, were deceased at follow-up, had a form of cognitive impairment, had bilateral or pathological fractures, had combined fractures of the same leg, suffered secondary injuries to the same knee during follow-up or had incomplete or inaccurate contact data.

### 2.2. Fracture Treatment

Initial imaging included conventional radiographs and computed tomography (CT). A conservative treatment approach was selected for patients with non- or minimally displaced fractures. This treatment strategy consisted of immobilization with an adjustable hinged knee brace, non-weight-bearing for a period of six weeks, and routine radiographic monitoring during follow-up.

Surgical intervention was tailored based on the complexity of the fracture, the condition of the surrounding soft tissue, and individual patient characteristics. In cases involving open fractures, extensive soft-tissue injury such as compartment syndrome, neurovascular compromise, or significant fracture displacement, temporary external fixation was employed for initial stabilization.

Minimally displaced unicondylar fractures were treated via screw fixation. More complex fracture patterns were managed through open reduction and internal fixation (ORIF) using locking compression plates (3.5 mm LCP, DePuy Synthes, Solothurn, Switzerland) via anterolateral, posterolateral, posteromedial, or anteromedial approaches, depending on the fracture configuration. Bicondylar fractures with severe comminution typically required dual plating (3.5 mm one-third tubular plate/LCP, DePuy Synthes, Solothurn, Switzerland), which was performed in a staged manner to reduce the risk of soft tissue complications. Subarticular bone defects were augmented with either allograft (DIZG, Berlin, Germany) or synthetic bone substitute (ChronOs, Arthrex, Naples, FL, USA). In cases of simple fractures with good soft-tissue conditions, arthroscopically assisted reduction and internal fixation (ARIF) was employed.

Postoperative care included limiting weight-bearing to 15 kg and restricting range of motion using an adjustable hinged knee brace for the first six weeks, followed by a stepwise increase in load and mobility. Hardware removal was considered once bone consolidation was confirmed radiographically, in response to local discomfort, or based on patient preference.

### 2.3. Outcome Measures

Patients included in the study received a standardized questionnaire to evaluate their participation in work and sports before and after TPF.

Patients categorized their previous occupation as “mostly sedentary”, “mostly standing and walking”, or “physically demanding”. The ability to return to the same job and the duration of work incapacity were assessed. RTW was defined as the time interval from injury (or surgery) to the resumption of work, either partially or fully.

Sports activities before and after TPF were recorded and classified based on physical intensity into “low-impact”, “intermediate-impact”, and “high-impact” sports, as previously described (Table 1) [14]. The competition level was also assessed, distinguishing between “recreational”, “competitive”, and “professional” sports. In addition, the number of weekly training sessions was documented. RTS was defined as the time interval from injury (or surgery) to the resumption of regular physical activity at any intensity and level.

Regarding posttraumatic or postoperative sport intensity and competition level, the highest level reached after injury was recorded, rather than the current level at follow-up, due to the variable follow-up duration.

Demographic information, injury mechanisms, and treatment details were gathered from medical records. The trauma mechanisms were divided into low-energy injuries, such as falls from a low height, and high-energy injuries, including falls from significant heights, bicycle accidents, skiing and other sports-related incidents, motorcycle crashes, and other traffic accidents. Fractures were categorized based on the Schatzker classification through an evaluation of radiological images.

### 2.4. Statistical Analysis

Statistical analysis was conducted using SPSS Statistics (Version 29.0, IBM Corp., Armonk, NY, USA).

Because of clear differences in injury severity, based on the respective indications for surgical or conservative treatment, and the unequal group sizes, this study did not aim to statistically compare the two treatment groups. Instead, a descriptive analysis was carried out, and the results are reported separately for each group.

Categorical data were summarized as absolute numbers and percentages. For continuous variables with a normal distribution, mean values and standard deviations (SD) were reported, whereas ordinal variables and those with a skewed distribution were described using the median (Md) and interquartile range (IQR). The normality of data distribution was assessed graphically and by use of the Kolmogorov–Smirnov test.

Time-to-event outcomes (RTW and RTS) were analyzed descriptively using Kaplan–Meier curves.

To evaluate potential factors influencing the duration of RTW and RTS, variables such as age, body mass index (BMI), American Society of Anesthesiologists (ASA) risk classification, work and sports intensity, time from injury to surgery, and surgical duration were analyzed using either the Pearson correlation coefficient or Spearman’s rank correlation coefficient. Normally distributed, continuous variables were analyzed using Pearson’s correlation coefficient, whereas ordinal or non-normally distributed variables were tested using Spearman’s rank correlation coefficient.

Return times were also compared between subgroups—male vs. female, smokers vs. non-smokers, high-energy vs. low-energy trauma, unicondylar fracture vs. bicondylar fracture, use of an external fixator vs. no external fixator, ARIF vs. ORIF, and implant removal vs. no implant removal—using the Mann–Whitney U test or an independent samples *t*-test. The Wilcoxon test was used to compare the number of sports and weekly workouts before and after TPF. A *p*-value of <0.05 was considered statistically significant.

## 3. Results

### 3.1. Demographics and Injury Mechanism

**Figure 1:**
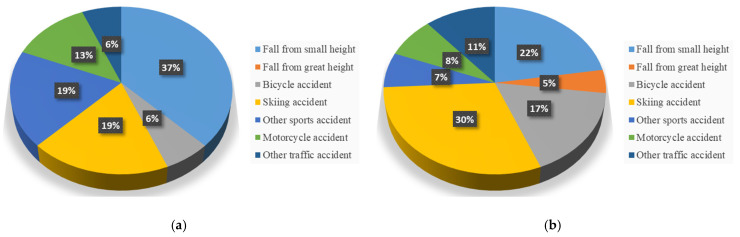
Trauma mechanisms of surgical (**a**) and conservative (**b**) treatment.

A total of 147 patients met the inclusion criteria, of whom 105 (71%) completed the follow-up. Among them, 89 patients (85%) received surgical treatment, while 16 (15%) were managed conservatively. An overview of the demographic data and fracture characteristics is provided in Table 2. 69 surgically treated patients (78%) and 10 conservatively treated patients (63%) sustained a high-energy trauma, while 48 (54%) and 7 (44%) were injured during sports, respectively (Figure 1).

### 3.2. Surgical Treatment

Surgical treatment was performed at a median of 5 days (IQR 3–9 days) after trauma. A temporary external fixator was used in 9 cases (10%), including one case of an open fracture classified as Gustilo and Anderson grade II. The most commonly used surgical approach was the anterolateral approach in 49 cases (55%), followed by the posteromedial approach in 7 cases (8%) and the anteromedial approach in 5 cases (6%). A combined medial and lateral approach was applied in 10 cases (11%). Minimally invasive percutaneous screw osteosynthesis was performed in 16 cases (18%), while ARIF was used in 18 cases (20%). ORIF with a single locking plate was the most frequent procedure, applied in 51 cases (57%), whereas double plating was required in 10 cases (11%). Screw-only osteosynthesis was performed in 28 cases (32%). Bone grafting for subarticular defects was necessary in 34 cases (38%). The median operative time was 121 min (IQR 80–187 min). 51 (57%) patients underwent removal of osteosynthetic material after a median of 1.3 years (IQR 1–2 years).

### 3.3. Return to Work

In the surgical treatment group, 75 patients (84%) were employed at the time of injury. Fifty-four of these patients (72%) described their occupation as “mostly sedentary”, 13 (17%) as “mostly standing and walking”, and 8 (11%) as “physically demanding”. All employed patients were able to return to their previous profession after 11.3 ± 9.5 weeks.

**Figure 2:**
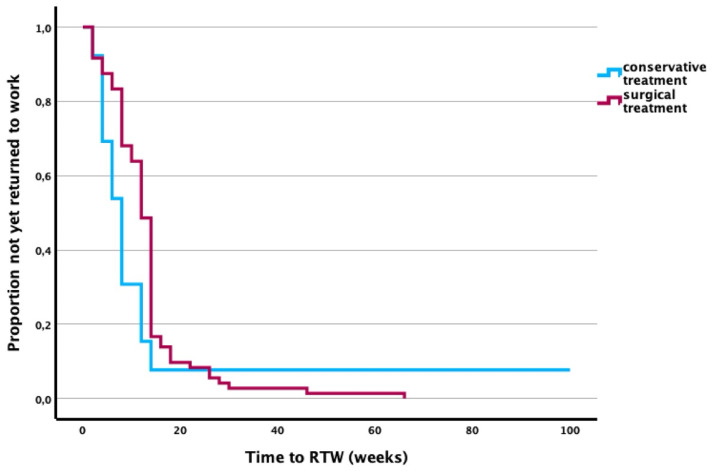
Kaplan–Meier curves showing time to RTW after TPF by treatment type.

In the conservative treatment group, 14 patients (88%) were employed. Of these, 8 (57%) reported having a “mostly sedentary” job, 5 (36%) a “mostly standing and walking” occupation, and 1 (7%) a “physically demanding” profession. 13 of the employed patients (93%) returned to their previous job after an average of 6.5 ± 4.2 weeks (Figure 2).

Regarding influencing factors on the duration of work incapacity, longer operation time was the only significant factor in the surgically treated group. In the conservatively treated group, higher age, ASA classification, and physical work intensity were significantly associated with prolonged work incapacity. Details are presented in Table 3.

In the surgical treatment group, subgroup analysis revealed a significantly shorter time to RTW for patients with high-energy trauma compared to those with low-energy trauma (10.4 ± 9.2 weeks vs. 14.9 ± 10 weeks, *p* = 0.019). In addition to that, a faster RTW was observed in patients with unicondylar fractures compared to those with bicondylar fractures (9.1 ± 5.5 weeks vs. 15.8 ± 13.7 weeks, *p* = 0.025). Furthermore, patients who underwent surgery without a temporary external fixator returned to work earlier than those who received external fixation (9.8 ± 5.7 weeks vs. 25.9 ± 21.4 weeks, *p* = 0.019). Subgroup analyses revealed no significant differences between male and female patients, smokers and non-smokers, patients treated with ARIF versus ORIF, or those who underwent implant removal compared to those who did not.

Additionally, no subgroup differences were observed within the conservatively managed cohort.

### 3.4. Return to Sports

Among patients in the surgical treatment group, 81 individuals (91%) reported engaging in regular physical activity prior to injury. A total of 69 (85%) resumed sporting activities after a mean duration of 22.1 ± 17.9 weeks.

**Figure 3:**
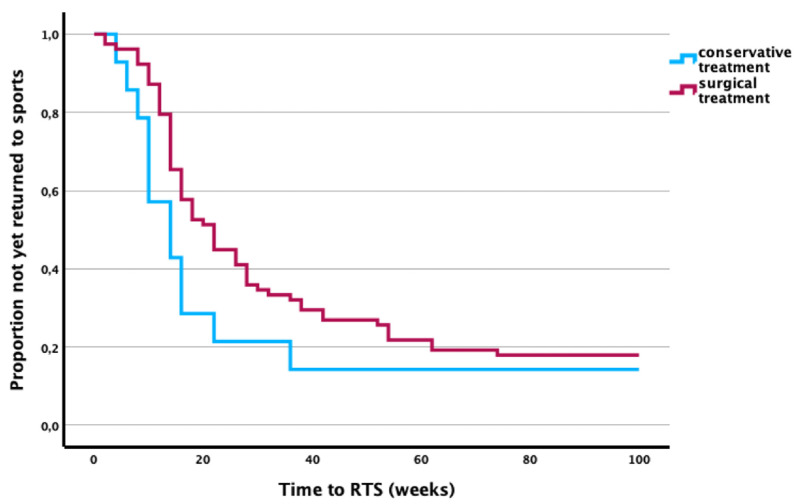
Kaplan–Meier curves showing time to RTS after TPF by treatment type.

In the conservative treatment group, 14 patients (88%) reported regular physical activity prior to injury, of whom 12 (86%) returned to sports after a mean duration of 12.2 ± 8.8 weeks (Figure 3).

Regarding influencing factors on the time to RTS, longer operation time was the only variable significantly associated with delayed return in the surgically treated group. No significant correlations were found in the conservatively treated group. Details are presented in Table 4.

Subgroup analysis in the surgical treatment cohort showed a faster RTS for male patients compared to female patients (17.9 ± 13.9 weeks vs. 24.9 ± 19.8 weeks, *p* = 0.036) and for patients without external fixation compared with those requiring external fixation (21.2 ± 17.5 weeks vs. 40.3 ± 17.9 weeks, *p* = 0.036). Patients who returned to the same or a higher level of physical sports intensity postoperatively required less time to do so compared to those who transitioned to a lower level (18.7 ± 13.8 weeks vs. 27.7 ± 22.3 weeks, *p* = 0.038). Subgroup analyses showed no significant differences between patients with high-energy and low-energy trauma, smokers and non-smokers, ARIF- and ORIF-treated individuals, unicondylar and bicondylar fractures, or between patients who underwent implant removal and those who did not.

Similarly, no significant subgroup differences were identified within the conservatively treated cohort.

**Figure 4:**
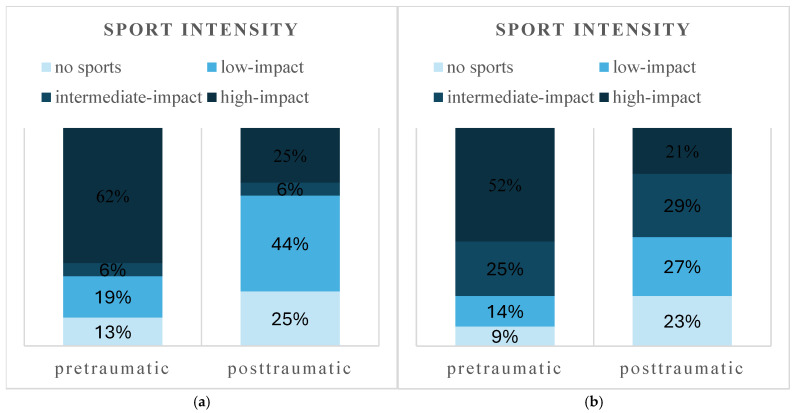
Pre- and posttraumatic sport intensity of surgical (**a**) and conservative (**b**) treatment.

**Figure 5:**
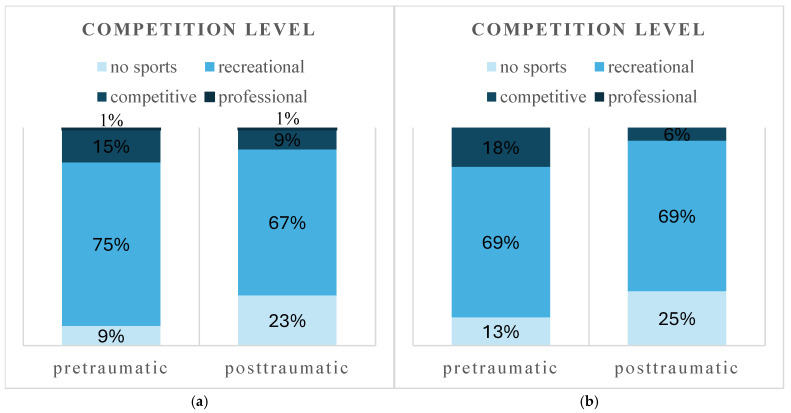
Pre- and posttraumatic competition level of surgical (**a**) and conservative (**b**) treatment.

A shift toward low-impact and recreational sports was observed in both the surgical and conservative treatment groups (Figure 4 and Figure 5). With surgical treatment, 52 patients (59%) returned to the same or higher level of sport intensity, and 70 patients (79%) returned to the same or a higher level of competition. With conservative treatment, 10 patients (63%) returned to the same sport intensity, and 12 patients (75%) returned to their previous competition level. Postoperatively, surgically treated patients participated in a reduced number of sports disciplines (Md 2, IQR 1–3 vs. Md 2, IQR 1–2, *p* < 0.001) and reported fewer training sessions per week (Md 2, IQR 1–3 vs. Md 2, IQR 1–3, *p* < 0.001).

Conservatively treated patients exhibited a similar pattern, with a lower number of sports disciplines practiced post-treatment (Md 1.5, IQR 1–3 vs. Md 1, IQR 1–2, *p* < 0.001) and fewer training sessions per week (Md 3, IQR 2–7 vs. Md 2.5, IQR 1–5, *p* < 0.001).

## 4. Discussion

The most important finding of the present study is that both surgical and conservative treatment approaches resulted in high return rates, with over 90% of patients returning to work and more than 85% resuming sports activities.

Surprisingly, all employed patients who underwent surgery were able to return to their previous occupations. Data on the duration of work incapacity are rarely reported, yet they are important for employers’ planning and are particularly essential for self-employed patients. Our analysis found that surgically treated patients returned to work after an average of 11 weeks. In comparison to previously published studies, our cohort demonstrated a higher RTW rate and a shorter duration of work incapacity. In a study by Dehoust et al. analyzing over 1.000 TPF from 2016 using registry data from the workers’ compensation insurance, the average duration of work incapacity was approximately 31 weeks. Furthermore, nearly 18% of patients received a disability pension within three years, corresponding to a reduction in earning capacity of at least 20%. The average total costs over three years, amounting to more than 23.000 euros per case for outpatient, inpatient, and rehabilitation expenses, underscore the socioeconomic significance of tibial plateau fractures [15]. Kraus et al. reported a median RTW time of 17 weeks, with 72% of patients returning to their previous job at the same capacity [3]. Van Dreumel et al. found that 77% of patients returned to work at least partly, with a median return time of approximately 24 weeks [4]. In our data collection, no distinction was made between partial and full RTW. This may have led to shorter reported return times if patients indicated the start of their reintegration as the time of return. Other possible reasons for the shorter duration of work incapacity and higher return rates in our cohort include the relatively young mean age, which was under 50 years, and the comparatively low injury severity, with two-thirds of patients sustaining unicondylar fractures. Additionally, the long follow-up period in our study may have captured delayed returns to work, which are often missed in studies with shorter follow-up durations.

Interestingly, patients with high-energy trauma returned to work faster, possibly because these injuries more often affect younger, more physically active individuals with higher rehabilitation potential. Factors such as bicondylar fracture patterns, the use of temporary external fixation, and longer operative times reflect greater injury severity. This increased severity is typically associated with higher morbidity, often due to the need for multiple surgical approaches. These aspects likely account for the association we observed between these factors and prolonged work incapacity. This is not surprising, as it is already well known that more complex injuries are associated with poorer functional outcomes [16,17].

Conservatively treated patients returned to work earlier, likely due to lower injury severity and the associated reduced treatment morbidity. The average duration of work incapacity in this group of just over 6 weeks reflects the period of partial weight-bearing recommended during rehabilitation, as basic mobility is a key requirement for many types of work. Nearly all patients with conservative treatment returned to their previous occupation, although comparable data are lacking in the existing literature. In contrast to the surgically treated group, older patients with more comorbidities, reflected by a higher ASA score, required more time to RTW. This is probably due to lower rehabilitation potential, as poorer outcomes in older and less healthy patients have been described before [18]. Similar to Kraus et al., we found an association between higher physical work intensity and a longer duration of work incapacity in the conservatively treated group [3].

Regarding RTS, approximately 85% of previously active patients resumed regular activity, regardless of treatment type. The mean time to return was 12 weeks in the conservative group and 22 weeks in the surgical group. A general shift toward low-impact and recreational sports was seen, regardless of treatment type, along with a reduction in the number of different sport disciplines and weekly training sessions. Although RTS was generally high, only about 60% of patients managed to resume sports at the same or a higher intensity level, and roughly three out of four returned to their previous or a higher competition level. A 2017 systematic review by Robertson et al. reported a 70% RTS after surgical treatment of tibial plateau fractures, with only 60% regaining their pre-injury level. However, only one study included provided return times, showing a median of 30 weeks [13]. More recent studies show variable results: O’Neill et al. found that only 46% of 90 surgically treated skiers returned to skiing, with half performing within one year, often at reduced frequency and on easier terrain [19]. Hap et al. reported return rates of 36% after surgery, with just 13% reaching pre-injury intensity and volume [5]. Kugelmann et al. observed a 53% return to athletic activity at a mean follow-up of 65 weeks [9]. Quintens et al., in a prospective study of 51 patients with posterior column involvement, found that 68% returned to sport partially after about 33 weeks and fully after 46 weeks, though over half reduced their activity frequency. Patients engaging in low-impact sports were significantly more likely to return than those in high-impact disciplines [11].

Similar to RTW, conservatively treated patients showed an earlier RTS compared to surgically treated patients, and we observed higher overall return rates and shorter return times than those reported in comparable literature. The factors previously discussed likely explain these findings here as well. In the context of RTS, a distinction is commonly made between return to participation, RTS, and return to performance [20]. In our study, return time refers to the point when patients resumed regular physical activity, matching the definition of return to participation. This stage typically occurs earlier than RTS or return to performance. Comparing our results to other studies is challenging, as many do not clearly define what is meant by RTS.

We did not investigate the reasons why patients reduced their sports intensity and volume after the injury, although this is a commonly observed phenomenon following tibial plateau fractures. Physical factors such as knee pain, limited range of motion, and instability likely play an important role in this context [11]. However, psychological factors, such as fear of reinjury or compromising the surgical outcome, should not be underestimated [5,11]. The importance of psychological factors in returning to sports is well known from other knee joint injuries, particularly from the extensively studied anterior cruciate ligament (ACL) injury [21,22,23]. In addition, due to the long follow-up period, patients were on average nearly ten years older at the time of follow-up than at the time of injury. As higher age is generally associated with a reduced activity level, this may also have contributed to the observed decline in sports participation [24].

Male sex and returning to the same or a higher level of sports intensity after surgery were associated with a faster RTS. A similar association was reported by O’Neill et al., where patients who returned to skiing were more often male and had sustained their injury while skiing [19]. Male patients may return to sports faster because they tend to be more impatient, have higher self-efficacy, and show greater confidence during recovery [25]. Additionally, patients who returned to the same or a higher level of sports intensity may have recovered faster because they were physically fitter before the injury, had clear goals and stronger motivation during rehabilitation, and in some cases, better access to physiotherapy or training support [22]. Furthermore, external fixation and longer surgery time were linked to a slower RTS. This likely shows again that more severe injuries lead to a longer recovery, just as seen with RTW.

The retrospective design represents a primary limitation of this study. Patients were asked to recall their physical activity before the injury, which in many cases dated back several years, increasing the risk of recall bias. The long follow-up period offers clear advantages but also presents some limitations, particularly in evaluating sport intensity and competition level. Factors such as the relatively high risk of post-traumatic osteoarthritis after tibial plateau fractures [26] and the increasing age of patients over time may have led to reduced sports activity over time. Therefore, this study assessed the highest level of sports activity reached after surgery rather than the current activity level. The long follow-up also made it difficult to control for all factors influencing the outcomes. Therefore, the identified associations should not be seen as the only influences. In addition, subgroup analyses were performed to provide descriptive insights into potential associations between injury and treatment characteristics and functional recovery. As the study was exploratory in nature, these analyses were not adjusted for multiple testing, and the results should therefore be interpreted with caution. With a complete follow-up rate of 71%, there is a potential for selection bias, as patients with better outcomes may have been more likely to participate. A systematic comparison of baseline data between responders and non-responders was not performed, which may have contributed to this bias. Lastly, this study was not designed to compare surgical and conservative treatment modalities, as the two subgroups differed substantially in both injury severity and sample size. Therefore, the results are presented descriptively and separately for each group. Despite the small number of conservatively treated patients, these findings still provide valuable insight, particularly given the lack of existing literature on RTW and RTS following non-operative management of tibial plateau fractures. In addition to that, strengths of the study include the long follow-up, the analysis of return times, and the detailed evaluation of associated factors. Treatment of all patients at a single level I university trauma center allowed for a consistent treatment approach and good comparability across the cohort.

## 5. Conclusions

This study demonstrates high RTW and RTS rates within the first six months following both surgical and conservative treatment of tibial plateau fractures. A general shift toward low-impact and recreational sports is common, along with a reduction in the number of sports’ disciplines and weekly training sessions. Patients requiring surgical treatment, especially those with complex fracture patterns, temporary external fixation, and longer operative times, generally experience longer recovery periods. Additionally, factors such as sex and the physical demands of work and sports influence the speed of recovery. These insights support more accurate patient counseling, helping to set realistic expectations to improve patient satisfaction, enhance therapy adherence, and provide greater planning certainty for both patients and clinicians.

## Figures and Tables

**Table 1:** Sports intensity.

Low-Impact	Intermediate-Impact	High-Impact
Bowling, Curling, Cycling, Exercise Walking, Golfing, Gymnastic Riding, Stretching, Swimming, Motorcycle driving	Aerobic, Dancing, Hiking, Musculation/Fitness, Rowing, Sailing, (Water) Gymnastics, Yoga	Badminton, Basketball, Cross Country Skiing, Downhill Skiing/Snowboarding, Fistball, Handball, Hockey, Inline Skating, Jogging, Martial Arts, Mountain Climbing, Paintball, Paragliding, Soccer, (Table-) Tennis, Track and Field, Trampolining, Volleyball, Wind/Kite surfing

**Table 2:** Demographic data and fracture characteristics.

	Surgical Treatment			Conservative Treatment		
	*n* (%)	*M* (*SD*)	*Md* (*IQR*)	*n* (%)	*M* (*SD*)	*Md* (*IQR*)
Male sex	39 (44)			8 (50)		
Age (years)		49.8 (12.5)			52.3 (17.6)	
BMI (kg/m^2^)		24.4 (3.4)			23.4 (3.4)	
Smokers	10 (11)			5 (31)		
ASA risk classification						
I	62 (70)			11 (69)		
II	24 (27)			5 (31)		
III	3 (3)			0 (0)		
Schatzker classification						
I–III	61 (69)			16 (100)		
IV–VI	28 (31)			0 (0)		
Follow-up (years)			10.5 (9–13)			10.2 (9–12)

*M* = mean; *SD* = standard deviation; *Md* = median; *IQR* = interquartile range; BMI = Body Mass Index; ASA = American Society of Anesthesiologists.

**Table 3:** Correlational Relationships involving duration of work incapacity.

	Duration of Work Incapacity			
	Surgical Treatment		Conservative Treatment	
	Pearson r/ Spearman’s ρ	*p*-Value	Pearson r/ Spearman’s ρ	*p*-Value
Age	0.059 *	0.623	0.632 *	0.020
BMI	0.188 *	0.110	−0.128 *	0.678
Operation time	0.492 *	<0.001	-	-
Time trauma-surgery	0.157 *	0.186	-	-
ASA risk classification	0.058 **	0.629	0.700 **	0.011
Work intensity	0.077 **	0.522	0.577 **	0.039

BMI = Body Mass Index; ASA = American Society of Anesthesiologists; * Pearson r; ** Spearman’s ρ.

**Table 4:** Correlational Relationships involving the time to return to sports.

	Time to Return to Sports			
	Surgical Treatment		Conservative Treatment	
	Pearson r/ Spearman’s ρ	*p*-Value	Pearson r/ Spearman’s ρ	*p*-Value
Age	0.073 *	0.548	0.100 *	0.757
BMI	−0.110 *	0.367	0.420 *	0.174
Operation time	0.345 *	0.004	-	**-**
Time trauma-surgery	−0.157 *	0.197	**-**	-
ASA risk classification	0.053 **	0.666	0.151 **	0.657
Physical sports intensity	−0.162 **	0.675	0.183 **	0.570
Competition level	0.003 **	0.485	−0.141 **	0.662

BMI = Body Mass Index; ASA = American Society of Anesthesiologists; * Pearson r; ** Spearman’s ρ.

## Data Availability

The raw data supporting the conclusions of this article will be made available by the authors on request.
